# Indoleamine 2,3-Dioxygenase Immune Status as a Potential Biomarker of Radioiodine Efficacy for Advanced Distant Metastatic Differentiated Thyroid Cancer

**DOI:** 10.3389/fonc.2022.871792

**Published:** 2022-07-18

**Authors:** Liang Shi, Rui Duan, Qiong Jia, Wenyu Wu, Jianming Zhou, Shaohua Li, Hao Zhang, Xue Xue

**Affiliations:** ^1^ Department of Nuclear Medicine, Nanjing First Hospital, Nanjing Medical University, Nanjing, China; ^2^ Department of Neurology, Nanjing First Hospital, Nanjing Medical University, Nanjing, China; ^3^ Department of Oncology, Nanjing First Hospital, Nanjing Medical University, Nanjing, China; ^4^ Department of Nuclear Medicine, Affiliated Hospital of Jiangsu University, Zhenjiang, China; ^5^ Department of Emergency, Affiliated Hospital of Jiangsu University, Zhenjiang, China

**Keywords:** differentiated thyroid cancer, radioiodine, indoleamine 2, 3-dioxygenase (IDO), immune suppression, predictive factors

## Abstract

**Purpose:**

Host immunity influences the impact of cancer therapy but the effect of immune status in radioiodine (RAI)-treated differentiated thyroid cancer (DTC) remains obscure. Here we investigated indoleamine 2,3-dioxygenase (IDO) activity as a biomarker of response to RAI in patients with distant metastatic DTC (dmDTC).

**Methods:**

Patients with dmDTC receiving RAI were evaluated for serum IDO activity (kynurenine and kynurenine:tryptophan ratio) at baseline and 3 months after RAI. The optimal cut-off value for these biomarkers to predict response was established by receiver operating characteristic analysis. The relationship between disease outcomes, overall survival (OS) and progression-free survival (PFS), and IDO activity levels was studied.

**Results:**

Higher baseline kynurenine:tryptophan ratio (>2.46) was correlated with poorer RAI response as well as shorter median PFS (45 mo versus not reached, *p*=0.002) and OS (78 mo versus not reached, *p*=0.035). High baseline kynurenine:tryptophan ratio was also correlated with a reduced number of tumor-infiltrating lymphocytes. Higher post/pre-kynurenine ratio (>1.69) was associated with survival endpoints: shorter median PFS (48 mo versus not reached, *p*=0.002) and OS (68 mo versus not reached, *p*=0.010). Favorable baseline and favorable change corresponded with better PFS and OS.

**Conclusions:**

Our results suggest that RAI also alters IDO activity in dmDTC patients. IDO activity could predict progression and survival outcomes for advanced dmDTC patients. Serum IDO biomarker levels could be used to select dmDTC likely to benefit from RAI therapy, although further studies are necessary.

## Introduction

Differentiated thyroid cancer (DTC) is the most common histological and least-aggressive type of thyroid cancer (1). However, 10%–20% of DTC patients develop distant metastatic disease, which is the most frequent cause of disease-specific death (2, 3). Distant metastatic DTC (dmDTC) patients require radioiodine (RAI) therapy at least once during their disease course. Although some patients with iodine-avid distant metastases may benefit from RAI therapy, this therapy is rarely curative (4).

RAI has been reported to activate the host immune system by reducing the secretion of Th2 cytokines [interleukin (IL)-4, IL-5, and IL-13] which might lead to tumor immune escape (5, 6). Therefore, in addition to direct DNA damage and tumor cell death, RAI might enhance antitumor immunity by those immunomodulatory factors in the tumor microenvironment (TME) (7). On the contrary, multiple immune suppressive factors are also activated by radiotherapy which results in aggressive and radiotherapy-resistant tumors with a poor clinical outcome (8). However, the relationship between RAI and blood biomarkers of immune function is not well understood.

Indoleamine-2,3-dioxygenase (IDO) is an intercellular enzyme that catalyzes conversion of tryptophan into kynurenine (9). IDO is expressed in a variety of different malignancies, and currently known as a cancer-related immunosuppressor (10, 11). IDO inhibits T cells by tryptophan depletion in the TME, which activates the amino acid–sensitive general control nonderepressible 2 stress-kinase pathway and causes cell cycle arrest and anergy induction in responding T cells (12). On the other hand, kynurenine, the product of IDO, enters natural killer (NK) cells *via* the aryl hydrocarbon receptor on the surface of NK cells. Kynurenine then decreases NK cells cytotoxicity in TME by inhibiting expression of NK-activating receptors, such as natural killer group 2D and NKp46, *via* signal transducer and activator of transcription (STAT)1 and STAT3 pathways (13). Furthermore, kynurenine and its downstream metabolites promote potent tumor immunosuppression *via* activation or differentiation of regulatory T (Treg) cells and effector T cells (14, 15). High IDO expression is associated with poor clinical outcome in diverse types of solid tumors, including thyroid cancer (16, 17). However, details of IDO activity in DTC have not been fully explored. The role of IDO activity as a biomarker for treatment outcomes and the prognostic significance in dmDTC treated with RAI remains unknown.

In this study, we evaluated the association between blood IDO activity levels and clinical benefit in advanced dmDTC patients treated with RAI. We examined differences in IDO biomarkers, including serum kynurenine and the kynurenine:tryptophan (K/T) ratio, before and 3 months after RAI. We then investigated whether IDO dynamics could represent a potential predictive biomarker for response to RAI. In addition, the association between circulating levels of IDO and overall survival (OS) and progression-free survival (PFS) in patients with dmDTC was studied.

## Materials and Methods

### Patients and Treatment

Patients with advanced dmDTC who received RAI therapy between January 2010 and June 2020 were identified from routine patient documentation at the Department of Nuclear Medicine, Nanjing First Hospital or the Affiliated Hospital of Jiangsu University. All patients signed a written informed consent prior to blood sampling according to the Declaration of Helsinki. This retrospective study was approved by the Institutional Ethics Committees of Nanjing First Hospital and the Affiliated Hospital of Jiangsu University. Baseline fasting blood samples and clinical characteristics of all dmDTC patients were obtained at the time of initial presentation at the hospital for the first cycle of RAI treatment.

### Therapeutic Approach and Follow-Up

All patients withdrew levothyroxine and began a low-iodine diet for 3–4 weeks before ^131^I treatment (thyroid-stimulating hormone reached 85.03 ± 35.37 uIU/mL). The first dose of oral ^131^I was 150–250 mCi (5.55–9.25 GBq). ^131^I whole body scan (WBS) was performed 4 days later. Patients with no ^131^I avid metastasis would be excluded. RAI therapy was repeated if the patient benefited from it, until complete remission or ^131^I inavidity on WBS. The treatment interval varied from 6 to 12 months, and the treatment was repeated for 2–8 cycles. The cumulative activity of ^131^I ranged from 11.1 to 57.35 GBq. The follow-up period was 1–11 years with a median of 64 months. Patients were examined 1 month after RAI, and followed up approximately every 3 months during the first year, and every 6 months from the second year thereafter. Post-RAI fasting blood samples of patients were collected at 3 months after the first cycle of RAI.

### Efficacy Evaluation

Response to RAI was defined according to the Response Evaluation Criteria in Solid Tumors (RECIST) 1.1. Responders were defined as patients with partial and complete responses, and non-responders were patients who had stable or progressive disease. If the patient had no measurable lesions, the response evaluation was based on thyroglobulin (Tg), a DTC tumor biomarker when antithyroglobulin antibody was negative. Compared with pretreatment, patients with a reduction of >25% in Tg levels were considered responders.

### Immunohistochemistry

DTC samples were obtained from surgical patients who provided signed informed consent at Nanjing First Hospital, Nanjing, China and Affiliated Hospital of Jiangsu University, Zhenjiang, China. Immunohistochemistry was performed as described previously (18). For immunohistochemistry, a mouse monoclonal anti-CD3 (1: 500, BD Biosciences Pharmingen, San Diego, CA, United States) and a mouse monoclonal anti-CD8 (1:150, BD Biosciences Pharmingen) were used. CD3^+^ and CD8^+^ tumor-infiltrating lymphocytes (TILs) were counted in a microscopic field at ×200 in the three independent areas with the most abundant lymphocyte infiltration.

### Measurements of Serum Tryptophan and Kynurenine

L-Tryptophan and L-kynurenine (Sigma, St. Louis, MO, USA) were used to construct standard curves. L-tryptophan-d5 and L-kynurenine-d4 (Cambridge Isotope Laboratories, Xenia, OH, USA) were used as internal standards. Tryptophan and kynurenine were measured using ultra-high-performance liquid chromatography (UPLC)–tandem mass spectrometry (MS/MS) (ACQUITY UPLC I-Class/Xevo TQD IVD System, Waters, USA) as described previously (19). Then 400 μL internal standard working solution was added to 150 μL human serum and 150 μL acetonitrile. A series of calibration standard solutions contained a mixture of drug-free serum (150 μL), L-tryptophan/L-kynurenine (150 μL), and internal standard (400 μL) working solution. Then 400 μL supernatant was collected after centrifugation at 12 000 rpm for 10 min and evaporated to dryness in a vacuum centrifugal concentrator. The residual was dissolved by 120 μL 1.2% formic acid. The supernatant was collected after centrifugation at 12 000 rpm for 5 min and injected into the chromatographic system for further analysis.

Tryptophan and kynurenine were analyzed by multiple reaction monitoring mode of MS/MS in positive ion mode. The cone voltage was 15–24 V, collision energy was 22–35 eV and transitions were m/z 205.0→118.0 for L-tryptophan, m/z 209.0→145.98 for L-kynurenine, and m/z 210.03→150.07 for L-tryptophan-d5, m/z 213.01→98.01 for L-kynurenine-d4.

### Statistical Analysis

PFS was defined as the time from the start of RAI to documented evidence of progression or death; OS was measured from the start of RAI to the date of death from any cause. The association of IDO checkpoint with RAI treatment response and response rate was assessed by Mann–Whitney U test or chi-square test. Differences between pre- and post-treatment IDO checkpoints were analyzed using a paired t-test. The cutoffs for the prediction of IDO significant biomarker variables were determined by receiver operating characteristic (ROC) curve analysis using response as an event. Kaplan–Meier method and a log-rank test were applied to compare the survival difference between groups. Univariate analysis for progression and survival was performed by Cox proportional hazards regression model. Multivariate analysis by Cox proportional hazards regression model with 95% confidence interval (95% CI) was used to evaluate clinical variables with log-rank *p*<0.05 under univariate analysis as covariants. All statistical analyses were performed with IBM SPSS Statistics version 23.0 (Armonk, NY, USA). Scatter plot figures were generated using GraphPad Prism version 8 (La Jolla, CA, USA).

## Results

### Patient Characteristics

Of 182 dmDTC patients enrolled, 104 patients (70 female, 34 male) with good-quality samples available for IDO testing formed the primary study population. The age of these patients was 48.7 ± 11.5 years (range 8–72 years). Distant metastasis as the initial evaluation of DTC was discovered in 25 cases (24.0%), and the diagnosis of distant metastasis was established during subsequent follow-up in 79 cases (76.0%). The median age at diagnosis of distant metastasis was 47 ± 11 years (range, 8–70 years). Sixty-five patients were at least 45 years old and 39 were <45 years old at the time of diagnosis. The distribution of pathological types of DTC was as follows: papillary thyroid carcinoma (PTC) in 74 patients (71.2%) and follicular thyroid carcinoma in 30 patients (28.8%). Fifty-five (52.9%) cases had only lung metastasis; 24 (23.1%) only bone metastasis; and 25 (24.0%) either combined lung and bone or other site metastases. Among other site metastases, four patients had mediastinal metastasis, two pleural metastasis, and one liver metastasis. Symptoms or events (like fractures) associated with any-site metastases occurred in 25 cases. **Table 1** shows the demographic and clinical features of the patients.

**Table 1:** Comparison of Overall Survival and Progression Free Survival Based on Clinical Characteristics of dmDTC Patients.

Variables	Patients (n)	Overall Survival			Progression Free Survival		
		Hazard Ratio	(95% CI)	P-value	Hazard Ratio	(95% CI)	P-value
**Age at diagnosis (years)**							
<45	39						
≥45	65	3.45	(1.18-10.07)	0.023	2.06	(1.06–4.03)	0.034
**Gender**							
Female	70						
Male	34	1.35	(0.58–3.13)	0.491	1.12	(0.57–2.18)	0.745
**Extent of metastases**							
Lung only	55			0.042			0.049
Bone only	24	1.48	(0.48–4.54)	0.491	1.09	(0.50–12.38)	0.832
Combined and other sites	25	3.09	(1.26–7.58)	0.014	2.24	(1.13–4.43)	0.021
**First presentation**							
No	79						
Yes	25	2.76	(1.25–6.10)	0.012	1.69	(0.90–3.17)	0.101
**Histologic type**							
PTC	74						
FTC	30	2.59	(1.18–5.70)	0.018	2.35	(1.25–4.35)	0.008
**Symptoms or events**							
No	79						
Yes	25	1.35	(0.67–3.23)	0.34	1.67	(0.92–3.06)	0.094
**T stage**							
1-2	49						
3-4	55	1.36	(0.62–2.99)	0.45	1.17	(0.64–2.13)	0.607
**Positive lymphatic invasion**							
No	38						
Yes	66	1.03	(0.46–2.31)	0.94	0.59	(0.31–1.14)	0.115

CI, confidence interval; PTC, papillary thyroid carcinoma; FTC, follicular thyroid carcinoma.

### Prognostic Clinical Factors and Overall Outcomes

Median follow-up time was 64 (95% CI, 56–72) months for all patients. The median PFS was 61 (95% CI, 47–75) months. Univariate analysis showed that the age of onset for distant metastases (*p*=0.023), pathological type (*p*=0.018), extent of metastases (*p*=0.042), whether the distant metastases were the first presentation of DTC (*p*=0.012), were significant risk factors for OS. Patients with younger age at diagnosis, PTC, noncombined site of distant metastasis, and discovery of distant metastases during follow-up had better OS than patients with older age at diagnosis, follicular thyroid carcinoma, combined sites of distant metastases, and discovery of distant metastases at diagnosis or before surgery. However, only age (*p*=0.034), extent of metastases (*p*=0.049), and pathological type (*p*=0.008) were significant for PFS (**Table 1**). These clinical parameters were thus selected as associated covariants for further multivariate analysis of IDO checkpoint biomarkers.

### Dynamics of Kynurenine and K/T Ratio at Baseline and Post-RAI

**Figure 1:**
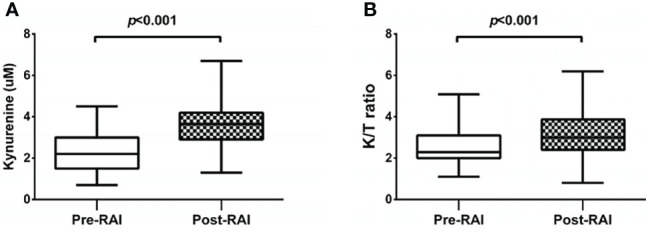
Changes of kynurenine concentrations **(A)** and the K/T ratios [Kynurenine (μM)/Tryptophan(μM) x 10^2^] **(B)** in serum samples at pre-RAI and post-RAI time points.

Serum kynurenine and K/T ratio fluctuated at two time points before and after RAI (**Figures 1A, B**). The mean kynurenine concentrations increased after RAI. The K/T ratio post-RAI was also significantly higher than that pre-RAI.

### Baseline IDO Biomarkers and Outcomes

**Figure 2:**
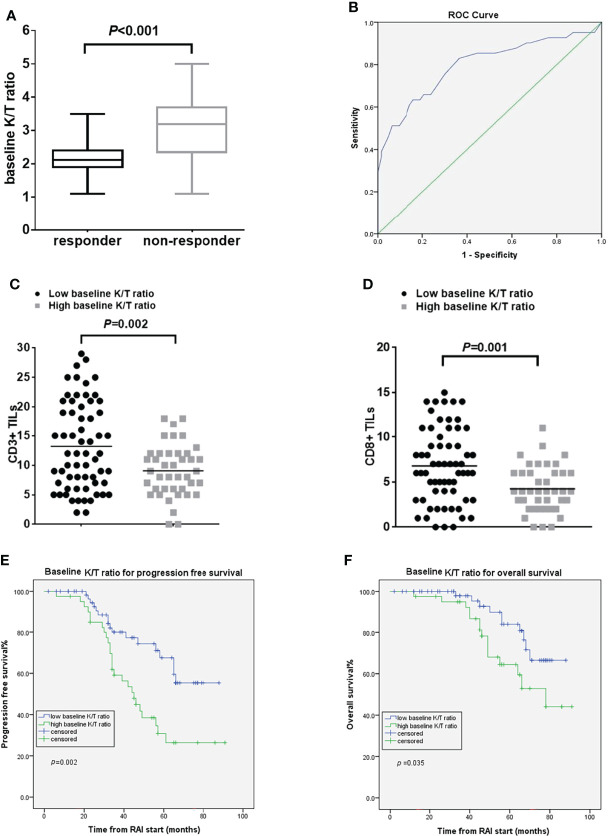
Pre-RAI K/T ratio and treatment outcomes. **(A)** the pre-RAI K/T ratio according to the response to RAI treatment **(B)** ROC curve for pre-RAI K/T ratio in dmDTC patients **(C)** association of baseline K/T ratio with the number of CD3^+^ TILs **(D)** association of baseline K/T ratio with the number of CD8^+^ TILs **(E)** PFS curves of the dmDTC patients according to pre-RAI K/T ratio **(F)** OS curves of the dmDTC patients according to pre-RAI K/T ratio.

The kynurenine levels at baseline did not differ between responders and non-responders. Baseline K/T ratio had a trend to be lower in responders than in non-responders [median 2.15 (range 1.10–3.50) vs. 3.10 (1.14–5.09) (*p*<0.001)] (**Figure 2A**). Using the ROC curve to determine the best value of baseline K/T ratio to predict response, we identified ≤2.46 (area under the curve 0.797, 95% CI 0.703–0.891, *p*<0.001) as the cutoff point that combined maximal sensitivity (65.9%) with best specificity (79.4%) (**Figure 2B**). Adopting the ROC-derived threshold, 63 (60.6%) patients were classified as having low baseline K/T ratio, while 41 (39.4%) were categorized as having high baseline K/T ratio. Response rate was significantly lower in patients with high compared with low baseline K/T ratio (13.5 vs. 47.1%, odds ratio 0.148, 95% CI 0.062–0.356, *p*<0.001) (**Supplementary Figure 1**). No correlation was found between baseline K/T ratio and tumor histological type. The tumors in patients with high baseline K/T ratio showed significantly lower CD3^+^ and CD8^+^ TIL numbers compared to the tumors in patients with low baseline K/T ratio (CD3^+^TILs: 9.07 ± 4.43 vs. 13.22 ± 7.46, *p* = 0.002; CD8^+^ TILs: 4.05 ± 2.26 vs. 6.29 ± 3.64, *p*=0.001) (**Figures 2C, D**; **Supplementary Figure 2**). Patients with high baseline K/T ratio had shorter PFS than those with low baseline K/T ratio [45 months vs. not reached, hazard ratio (HR) 2.50, 95% CI 1.35–4.63, *p*=0.002], and shorter median OS (78 months vs. not reached, HR 2.32, 95% CI 1.03–5.19, *p*=0.035) (**Figures 2E, F**). In the multivariate Cox regression analysis, elevated baseline K/T ratio correlated significantly with worse OS (HR 5.32; 95% CI, 1.96–14.44; *p*=0.001) and PFS (HR 3.75; 95% CI, 1.87–7.54; *p*<0.001) after adjusting for clinically significant factors (**Table 2**).

**Table 2:** Multivariate Analyses of Factors Predicting Overall Survival and Progression Free Survival.

	Overall Survival			Progression Free Survival		
Variables	Hazard Ratio	(95% CI)	P-value	Hazard Ratio	(95% CI)	P-value
**Age at diagnosis (years)**						
<45						
≥45	3.51	(1.19–10.35)	0.023	2.87	(1.42–5.78)	0.003
**Extent of metastases**						
Lung only			0.009			0.042
Bone only	0.639	(0.20–2.07)	0.491	0.69	(0.31–1.56)	0.375
Combined and other sites	3.73	(1.38–10.09)	0.010	2.02	(1.00–4.09)	0.050
**First presentation**						
No						
Yes	2.92	(1.25–6.81)	0.013	2.54	(1.30–4.96)	0.007
**baseline K/T ratio**						
Low						
High	5.32	(1.96–14.44)	0.001	3.75	(1.87–7.54)	0.000
**Post/pre-kynurenine ratio**						
Low			–			
High	–	–	–	2.76	(1.34–5.71)	0.006

CI, confidence interval; PTC, papillary thyroid carcinoma; FTC, follicular thyroid carcinoma

### Post-RAI IDO Biomarkers and Outcomes

**Figure 3:**
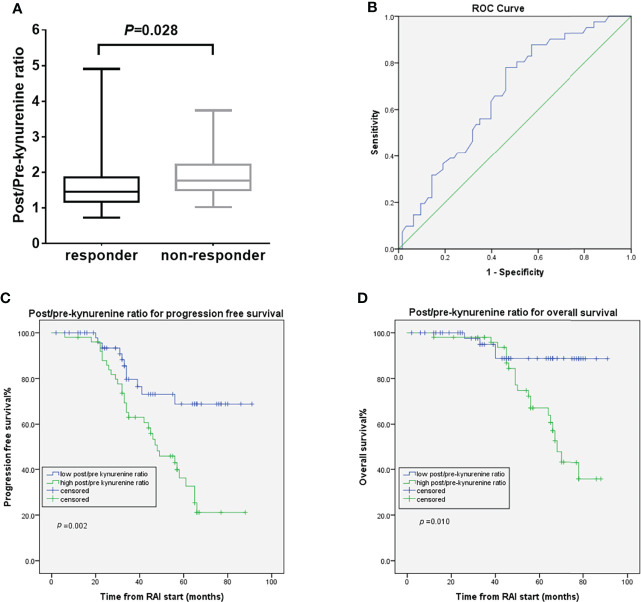
Post/pre-kynurenine ratio and treatment outcomes. **(A)** the post/pre-kynurenine according to the response to RAI treatment **(B)** ROC curve for post/pre-kynurenine in dmDTC patients **(C)** PFS curves of the dmDTC patients according to post/pre-kynurenine **(D)** OS curves of the dmDTC patients according to post/pre-kynurenine.

Neither the IDO biomarker (kynurenine or K/T ratio) levels post-RAI nor RAI-induced K/T ratio changes post-RAI differed between responders and non-responders. The RAI-induced post/pre-kynurenine ratio was significantly higher in non-responders compared with responders [median 1.95 μmol/L (range 1.67–4.91) vs. 1.64 μmol/L (range 0.73–2.07), *p*=0.028] (**Figure 3A**). By conducting ROC curve analyses, 1.69 (AUC 0.66, 95%CI 0.56–077, *p*=0.005) was calculated as the best cutoff for post/pre-kynurenine ratio to differentiate responders from non-responders (**Figure 3B**). The sensitivity and specificity of post/pre-kynurenine ratio in predicting therapy response were 56.1% and 60.3%, respectively. The response rate was lower in the high than in low post/pre-kynurenine ratio group (13.5 vs. 37.5%, odds ratio 0.319, 95% CI 0.140–0.726, *p*=0.006) (**Supplementary Figure 3**). No correlation was found between post/pre-kynurenine ratio and tumor histological type. High post/pre-kynurenine ratio correlated significantly with worse PFS (48 months vs. not reached, HR 2.90, 95% CI, 1.48–5.77, *p*=0.002) and poorer OS (68 months vs. not reached, HR 4.08, 95% CI, 1.40–11.92, *p*=0.010) (**Figures 3C, D**). The significance of post/pre-kynurenine ratio associated with OS in multivariate Cox regression model was lost when adjusting for clinically significant factors. Otherwise, in the multivariate Cox regression models for PFS, post/pre-kynurenine ratio remained an independently predictive factor (HR 2.76; 95% CI, 1.34–5.71; *p*=0.006; **Table 2**).

### Effects of Combined IDO Biomarkers on OS and PFS

**Figure 4:**
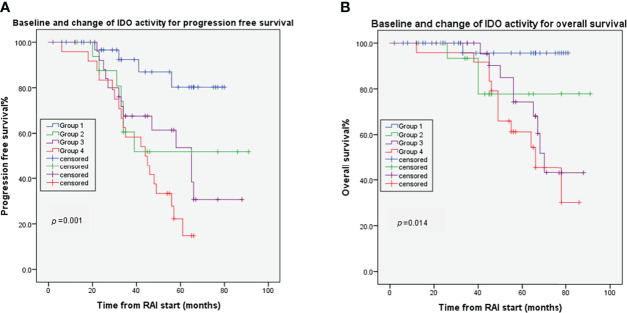
Combined effects of baseline Change of IDO on DTC tumor progression **(A)** and overall survival **(B)**. Group 1: patients with low baseline K/T ratio and low post/pre-kynurenine ratio; Group 2 patients with high baseline K/T ratio and low post/baseline kynurenine ratio; Group 3: patients with low baseline K/T ratio and high post/baseline kynurenine ratio; Group 4: patients with high baseline K/T ratio and high post/baseline kynurenine ratio.

Considering serum K/T ratio at baseline and RAI-induced post/pre-kynurenine ratio change, the 104 evaluable patients were classified into four groups. Group 1 included 36 (34.6%) patients with low baseline K/T ratio and low post/pre-kynurenine ratio. Group 2 included 17 (16.3%) patients with high baseline K/T ratio and low post/baseline kynurenine ratio. Group 3 included 27 (26.0%) patients with low baseline K/T ratio and high post/baseline kynurenine ratio. Group 4 included 24 (23.1%) patients with high baseline K/T ratio and high post/baseline kynurenine ratio. There were significant differences in PFS (*p*=0.001) (**Figure 4A**) and OS (*p*=0.014) (**Figure 4B**) for these four groups. The median OS and PFS were not reached after 64 months of median follow-up in Groups 1 and 2. Median OS and PFS were 70 months (95% CI 65–75) and 65 months (95% CI 46–84) in Group 3, and 66 months (95% CI 48–84) and 44 months (95% CI 31–57) in Group 4, respectively. Groups 3 and 4 had shorter OS than Group 1 (HR 9.284, 95% CI 1.18–73.32, *p*=0.035; HR 14.08, 95% CI 1.82–108.72, *p*=0.011, respectively). Groups 2–4 had poorer PFS than Group 1 (HR 3.93, 95% CI 1.14–13.44, *p*=0.029; HR 4.09, 95% CI 1.33–12.54, *p*=0.014; HR 7.39, 95% CI 2.49–21.92, *p*<0.001, respectively).

## Discussion

In the present retrospective study, we found that non-responders to RAI had higher serum K/T ratio at baseline compared to responders. Patients with low baseline K/T ratio achieved longer PFS and OS than those harboring high serum baseline K/T ratio.

We hypothesized that IDO activity could be useful to predict response to RAI in dmDTC patients, based on the immunoinhibitory properties of IDO and its function as a potent immune checkpoint in cancer. IDO is highly expressed in DTC tumor tissues (17). IDO activity, as reflected by serum kynurenine levels, was higher in PTC patients than that in healthy controls (20). IDO immune status was also higher in DTC patients with lung metastases than in patients without metastases (21). High expression of IDO in FTC-133 human thyroid cancer cells exerted a strong suppressive action on the proliferation of activated T lymphocytes (17). To the best of our knowledge, this study is the first study to show the predictive value of serum IDO activity biomarkers in patients with DTC.

In our analysis, baseline K/T ratio was an important prognostic marker for dmDTC patients treated with RAI. This is in agreement with previous studies in which baseline IDO activity might have served as prognostic factors treated with chemoradiation (22, 23). IDO controls complement-dependent enhancement of chemoradiation therapy against murine glioblastoma (24). Mouse knockout studies have indicated that IDO promotes breast tumor resistance to chemotherapy *via* immune escape from T-cell-dependent anti-tumor immunity (25). In this study, we analyzed the infiltration of TILs and evaluated the relationship between IDO activity and TIL counts. Interestingly, baseline K/T ratio was negatively related with CD3^+^ or CD8^+^ TILs. This is consistent with previous reports showing the immunosuppressive role of IDO enzyme in the malignant tumor disease (26, 27) Thus, our findings demonstrated that IDO activity is associated with the suppressed infiltration of T cells into the tumor tissues, which may contribute to poor response to RAI.

RAI causes DNA double-strand breaks, which can lead to efficient DTC cell killing. Tumor-derived IDO improves DNA damage repair and mediates resistance to chemoradiation in human cancer cells by a steady supply of nicotinamide adenine dinucleotide generated from the IDO downstream metabolic products (28). This is the evidence that IDO promotes cancer survival through another mechanism, independently of the immune system. Our preliminary data provide strong clinical confirmation of the preclinical studies concerning the role of IDO in DTC. More mechanistic investigations are required to confirm these findings.

Another major finding of our study was that RAI induced IDO activity change, reflected by post/pre-kynurenine ratio, was higher in RAI non-responders, with shorter OS and PFS. Most previous studies have shown the relationship between IDO activity at baseline and prognosis of patients with acute myeloid leukemia (29, 30), breast cancer (31), cervical cancer (32), lymphoma (33–35), colorectal cancer (36), glioma (37), lung cancer (22, 23, 38–41), and melanoma (42, 43) (**Table 3**). Only a few studies investigated the relationship between dynamic IDO activity change and outcome of tumor patients after radiotherapy or chemotherapy. One study reported that the K/T ratio was increased in non-small-cell lung cancer (NSCLC) patients after chemoradiation therapy and such an increased IDO activity portended worse OS and PFS (22). Elevated post/pre-kynurenine ratio in stage III NSCLC patients treated with chemoradiation had significantly worse OS (23). Higher serum kynurenine levels after radiotherapy were associated with worse OS in patients with newly diagnosed stage I/II NSCLC (38). As far as we know, there are no studies on the IDO levels in circulation in patients with advanced thyroid cancer. Our study demonstrated that the increase of IDO activity after RAI was related to therapy response in DTC. The possible reason might be that after massive radiosensitive tumor cells killing by ionizing irradiation, the proliferation of radioresistant tumor cells dominate the TME and present an immunosuppressive phenotype, or that RAI directly alters the biological behavior of tumor cells and leads to suppression of the anti-tumor microenvironment. These findings support that in addition to IDO activity at baseline as a prognostic biomarker for DTC patients, RAI therapy might affect host tumor immune microenvironment, modify the therapeutic response, and finally change the prognosis in DTC patients. Further studies are needed to explore the mechanisms of immunoregulation under RAI.

**Table 3:** Prognostic Value of IDO Activity in Different Cancer Types.

Cancer	N(adults)	Serum IDO Markers	Detection method	Prognostic value	Ref.
Acute myeloid leukemia	184	Kyn/Trp ratio	HPLC	Increased Kyn/Trp ratio associated with short OS	(29)
	48	Kyn concentration	HPLC	Increased Kyn levels associated with short OS	(30)
Breast cancer	32	Trp/Kyn ratio	HPLC	Lower Trp/Kyn ratio associated with shorter OS	(31)
Cervical cancer	251	Kyn/Trp ratio	MS	Increased Kyn/Trp ratio associated with short DFS	(32)
T-cell leukemia/lymphoma	96	Kyn/Trp ratio; Trp concentration	MS	Increased Kyn/Trp ratio and the levels of Kyn associated with short OS	(33)
Follicular lymphoma	110	Kyn/Trp ratio; Trp concentration	MS	Increased Kyn/Trp ratio and the levels of Kyn associated with short OS	(34)
Non-Hodgkin lymphoma	73	Trp concentration	HPLC	Lower Trp levels associated with shorter OS	(35)
Colorectal cancer	66	Trp concentration	HPLC	Lower Trp levels associated with liver metastases and reduced quality of Life	(36)
Glioma	33	Kyn/Trp ratio	HPLC	Increased Kyn/Trp ratio associated with short OS	(37)
Lung cancer	33	Kyn/Trp ratio	MS	Increased Kyn/Trp ratio after induction and chemoradiation therapy associated with short OS and PFS	(22)
	110	Kyn/Trp ratio; Kyn concentration	HPLC	Increased pre-treatment Kyn/Trp ratio and changes in the levels of Kyn after radiation associated with poor OS	(23)
	56	Kyn concentration	HPLC	Increased levels of Kyn post-radiation associated with poor OS	(38)
	104	Kyn/Trp ratio	HPLC	Increased Kyn/Trp ratio pre-treatment associated with short PFS; increased post/pre-Kyn : Trp ratio after radiation associated with OS	(39)
	123	Kyn/Trp ratio	MS	Increased Kyn/Trp ratio associated with short OS	(40)
	252	Kyn/Trp ratio	HPLC	Increased Kyn/Trp associated with decreased efficacy of chemotherapy	(41)
Melanoma	87	Kyn/Trp ratio	HPLC	Increased Kyn/Trp ratio associated with short OS	(42)
	186	Kyn concentration	HPLC	Increased levels of Kyn post-radiation associated with short OS	(43)

Kyn, kynurenine; Trp, tryptophan; HPLC, high performance liquid chromatography; MS, mass spectrometry; Ref., references; OS, overall survival; PFS, progression-free survival.

The capability of IDO to predict outcomes of RAI treatment was further confirmed by the combined use of baseline and the change of IDO activity after RAI. Patients who maintained low baseline K/T ratio and low post/pre-kynurenine ratio achieved better PFS and OS than those who maintained high baseline K/T or post/pre-kynurenine ratio. This clinical experience suggests that, on the one hand, the differences in baseline IDO activity of the dmDTC patients would represent individual immune status variation in tumor pathophysiological characteristics. Baseline IDO biomarker might be used to select suitable patients who may benefit from RAI treatment. On the other hand, the change in IDO activity after RAI therapy dynamically reflected the tumor response to RAI in biological behavior and immune environment, allowing for early detection of cases with acquired resistance to RAI.

Regarding RAI-treated DTC patients, Tg has been reported to have prognostic value. Unfortunately, patients with poorly differentiated thyroid carcinoma and those with positive anti-Tg antibody show decreased expression of Tg (44, 45). In those cases, patients cannot be evaluated with Tg (46). Evaluation of other available tissue biomarkers, such as sodium iodine transporter, requires adequate samples that can only be obtained in an invasive manner, and is hindered by spatial and temporal heterogeneity. These data underline the challenge to identify a reliable biomarker among the systemic immune components with a clear predictive value for DTC patients, since the development and maintenance of an immune microenvironment has shown clear associations with individual outcome (47).

This study had some limitations. First, the number of patients was limited, which may affect study results. Second, correlative immunological markers, such as forkhead box P3 expression and other T lymphocyte subset, were not measured, which may help to confirm the putative role of IDO in DTC patients. Third, the cutoff value of IDO biomarker was derived from analysis of the study population and thus needs to be validated in an external series of patients. Finally, IDO1 might act as an immunosuppressor in a context-dependent manner and its expression is induced by specific oncogenes (13). The association between serum IDO biomarker levels and mutations of clinical tumor driver genes involved in DTC initiation and progression was not evaluated in this study.

In conclusion, our study provides clinicians with an independent and significant prognostic biomarker in dmDTC patients treated with RAI. Serum IDO activity could represent a noninvasive dynamic biomarker that is available in every patient, demonstrating disease and evolution of tumor immune environment over time and enabling early detection of cases with immunobiological resistance to RAI. Finally, our findings, if confirmed, may reveal the development of novel multimodality clinical trials using anti-IDO agents that might improve the efficacy of RAI by blocking the potential immunosuppressive action of IDO.

## Data Availability Statement

The original contributions presented in the study are included in the article/**Supplementary Material**. Further inquiries can be directed to the corresponding authors.

## Ethics Statement

The studies involving human participants were reviewed and approved by the Institutional Ethics Committees of Nanjing First Hospital, the Institutional Ethics Committees of Affiliated Hospital of Jiangsu University. The patients/participants provided their written informed consent to participate in this study.

## Author Contributions

XX, LS, and HZ conceived and designed the study. XX and HZ supervised the study. LS and RD did the statistical analysis. QJ, WW, JZ, and SL contributed to acquisition, analysis, or interpretation of data. XX, LS, RD, QJ, and HZ drafted the manuscript. All authors revised the report and approved the final version before submission.

## Funding

This work was supported by grants from the National Natural Science Foundation of China (82001865), the National Thyroid Research Project for Chinese Young and Middle-aged Doctors (2020), the Clinical Research Project of Nanjing Medical University (NMUB2019169), the Natural Science Foundation of Jiangsu Province (BK20200145).

## Conflict of Interest

The authors declare that the research was conducted in the absence of any commercial or financial relationships that could be construed as a potential conflict of interest.

The reviewers XW and JZ declared a shared affiliation with the authors to the handling editor at the time of review.

## Publisher’s Note

All claims expressed in this article are solely those of the authors and do not necessarily represent those of their affiliated organizations, or those of the publisher, the editors and the reviewers. Any product that may be evaluated in this article, or claim that may be made by its manufacturer, is not guaranteed or endorsed by the publisher.

## Supplementary Material

Supplementary Figure 1 Response rate in patients with low and high pre-RAI K/T ratio groups.

Supplementary Figure 2 Representative images of CD3^+^ and CD8^+^ tumor-infiltrating lymphocytes in two different with DTC. (patient 1: non-responder;patient 2:responder). Scale bar, 50 μm.

Supplementary Figure 3 Response rate in patients with low and high post/pre-kynurenine groups.
