# Supplementary figures and images for: Indoleamine 2,3-Dioxygenase Immune Status as a Potential Biomarker of Radioiodine Efficacy for Advanced Distant Metastatic Differentiated Thyroid Cancer
Source: Front Oncol. 2022 Jul 18;12:871792. doi: 10.3389/fonc.2022.871792 (PMC9339611; doi:10.3389/fonc.2022.871792)

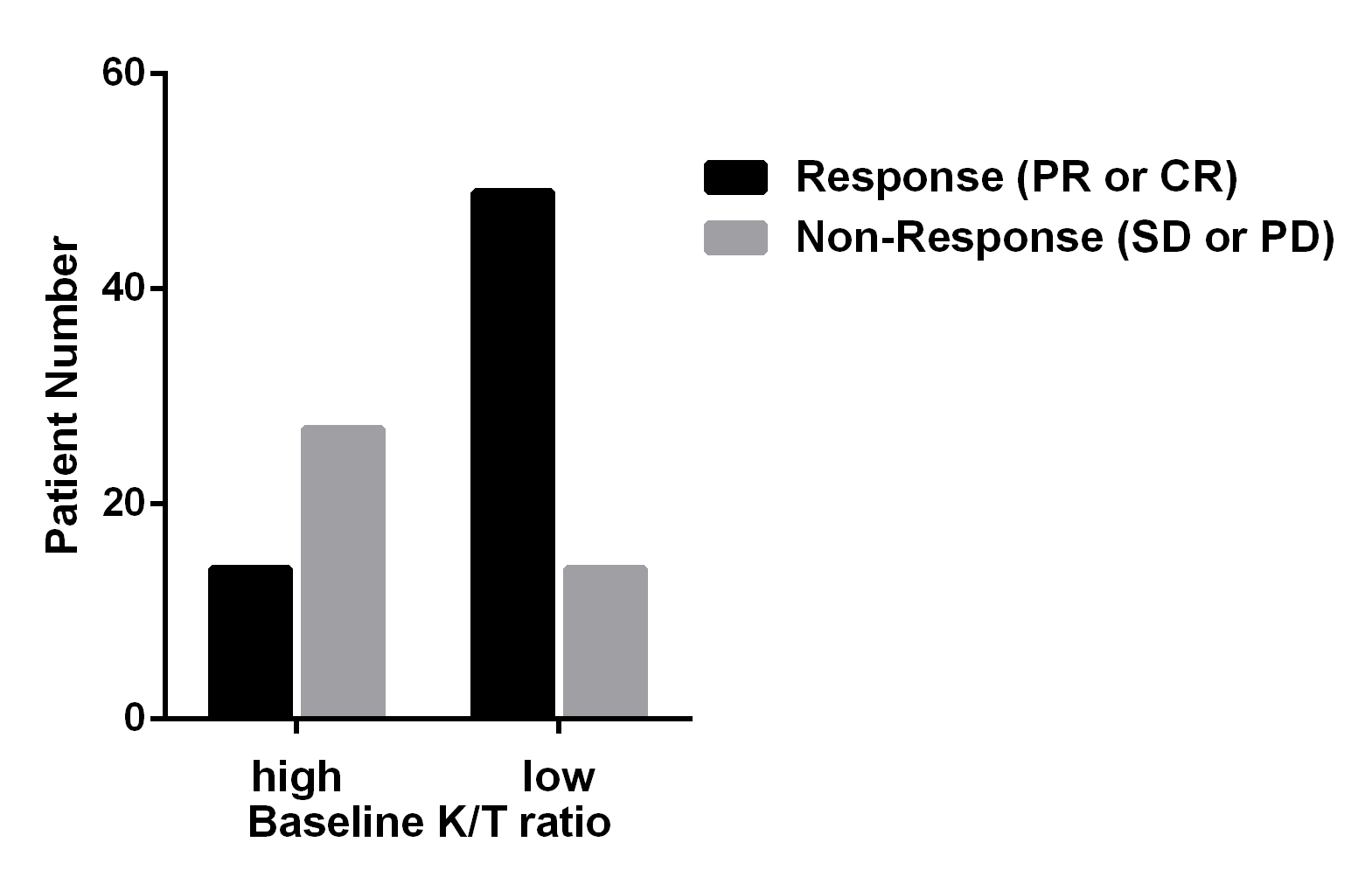

Supplement: Supplementary Figure 1 — Response rate in patients with low and high pre-RAI K/T ratio groups. [file Image_1.tif]

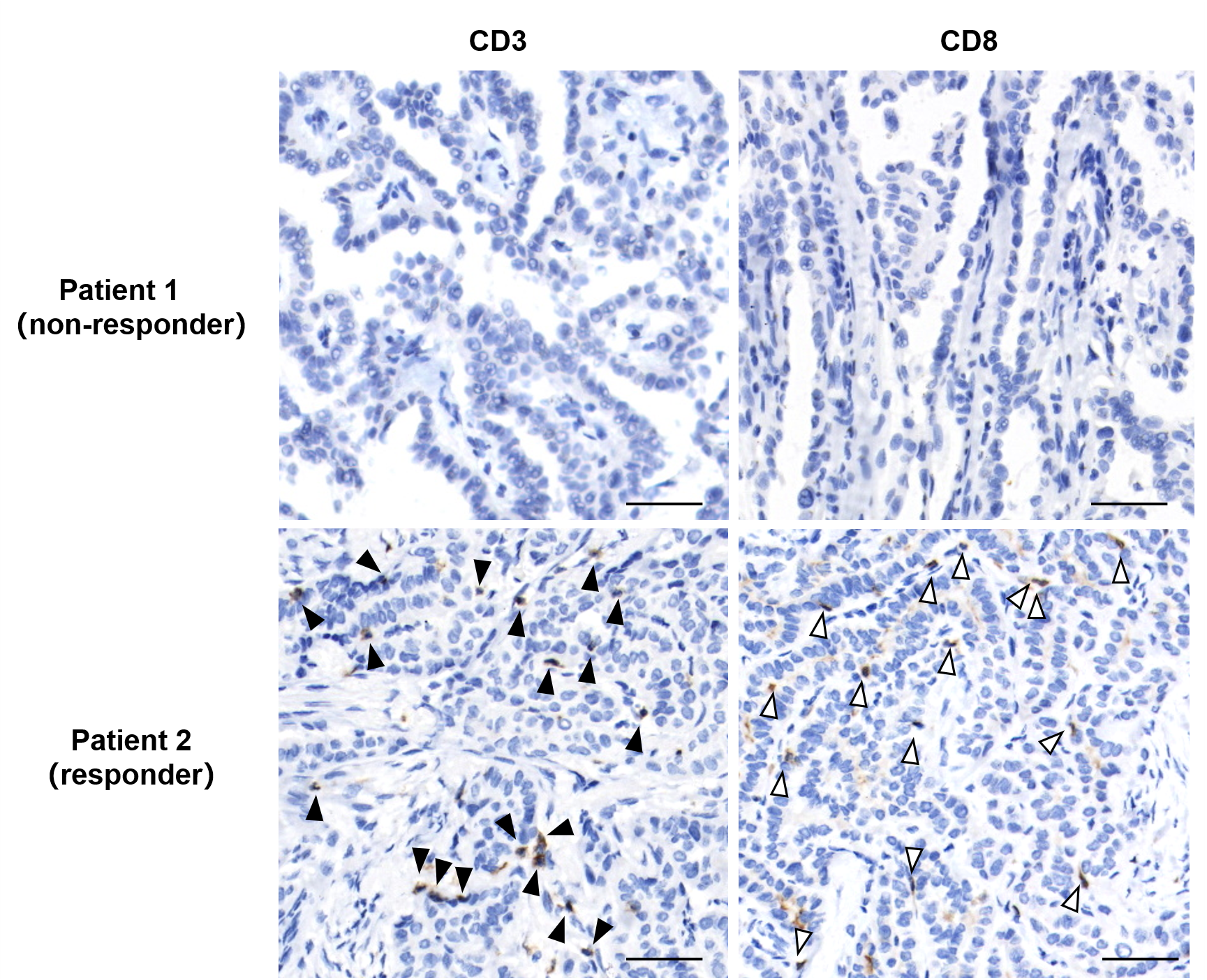

Supplement: Supplementary Figure 2 — Representative images of CD3+ and CD8+ tumor-infiltrating lymphocytes in two different with DTC. (patient 1: non-responder;patient 2:responder). Scale bar, 50 μm. [file Image_2.tif]
